# 4,4′,6,6′-Tetra-*tert*-butyl-2,2′-[1,2-phenyl­enebis(nitrilo­methyl­idyne)]diphenol acetone solvate

**DOI:** 10.1107/S1600536808003905

**Published:** 2008-02-13

**Authors:** Naser Eltaher Eltayeb, Siang Guan Teoh, Suchada Chantrapromma, Hoong-Kun Fun, Rohana Adnan

**Affiliations:** a School of Chemical Science, Universiti Sains Malaysia, 11800 USM, Penang, Malaysia; b Department of Chemistry, Faculty of Science, Prince of Songkla University, Hat-Yai, Songkhla 90112, Thailand; c X-ray Crystallography Unit, School of Physics, Universiti Sains Malaysia, 11800 USM, Penang, Malaysia

## Abstract

In the Schiff base mol­ecule of the title compound, C_36_H_48_N_2_O_2_·C_3_H_6_O, the central benzene ring makes dihedral angles of 46.64 (10) and 49.34 (10)° with the two outer benzene rings, and the two outer benzene rings form an angle of 39.13 (8)°. There are two intra­molecular O—H⋯N hydrogen bonds involving the two hydr­oxy groups, which generate *S*(6) ring motifs. In the crystal structure, the Schiff base mol­ecules are linked into a chain along the *a* axis by C—H⋯π inter­actions. The acetone solvent mol­ecules are attached to the chain *via* C—H⋯O hydrogen bonds.

## Related literature

For bond-length data, see: Allen *et al.* (1987 ▶). For ring-motifs, see: Bernstein *et al.* (1995 ▶). For biological activities of Schiff base compounds, see: Dao *et al.* (2000 ▶); Eltayeb & Ahmed (2005*a* ▶,*b* ▶); Karthikeyan *et al.* (2006 ▶); Sriram *et al.* (2006 ▶). For related structures, see: Eltayeb, Teoh, Chantrapromma *et al.* (2007 ▶); Eltayeb, Teoh, Teh *et al.* (2007*a* ▶,*b* ▶).
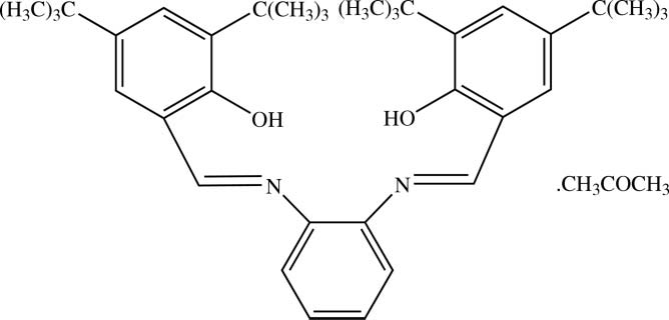


## Experimental

### 

#### Crystal data

C_36_H_48_N_2_O_2_·C_3_H_6_O
*M* *_r_* = 598.84
Triclinic, 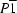

*a* = 10.0008 (2) Å
*b* = 12.0020 (3) Å
*c* = 17.0366 (4) Å
α = 82.068 (1)°
β = 86.320 (1)°
γ = 65.764 (1)°
*V* = 1846.76 (7) Å^3^
*Z* = 2
Mo *K*α radiation
μ = 0.07 mm^−1^
*T* = 296 (2) K
0.59 × 0.55 × 0.43 mm

#### Data collection

Bruker SMART APEX2 CCD area-detecto diffractometer
Absorption correction: multi-scan (*SADABS*; Bruker, 2005 ▶) *T*_min_ = 0.962, *T*_max_ = 0.972
26090 measured reflections
8384 independent reflections
5874 reflections with *I* > 2σ(*I*)
*R*_int_ = 0.022

#### Refinement

*R*[*F* ^2^ > 2σ(*F* ^2^)] = 0.060
*wR*(*F* ^2^) = 0.183
*S* = 1.04
8384 reflections
419 parameters
H atoms treated by a mixture of independent and constrained refinement
Δρ_max_ = 0.45 e Å^−3^
Δρ_min_ = −0.28 e Å^−3^

### 

Data collection: *APEX2* (Bruker, 2005 ▶); cell refinement: *APEX2*; data reduction: *SAINT* (Bruker, 2005 ▶); program(s) used to solve structure: *SHELXTL* (Sheldrick, 2008 ▶); program(s) used to refine structure: *SHELXTL*; molecular graphics: *SHELXTL*; software used to prepare material for publication: *SHELXTL* and *PLATON* (Spek, 2003 ▶).

## Supplementary Material

Crystal structure: contains datablocks global, I. DOI: 10.1107/S1600536808003905/ci2559sup1.cif

Structure factors: contains datablocks I. DOI: 10.1107/S1600536808003905/ci2559Isup2.hkl

Additional supplementary materials:  crystallographic information; 3D view; checkCIF report

## Figures and Tables

**Table 1:** Hydrogen-bond geometry (Å, °) *Cg*1 and *Cg*2 are the C1–C6 and C15–C20 ring centroids, respectively.

*D*—H⋯*A*	*D*—H	H⋯*A*	*D*⋯*A*	*D*—H⋯*A*
O1—H1*O*1⋯N1	0.89 (2)	1.76 (3)	2.5809 (19)	153 (3)
O2—H1*O*2⋯N2	0.87 (3)	1.78 (3)	2.5956 (19)	155 (3)
C7—H7*A*⋯O3	0.93	2.58	3.460 (6)	158
C30—H30*B*⋯O2	0.96	2.36	2.994 (3)	123
C31—H31*A*⋯O2	0.96	2.34	2.989 (3)	124
C34—H34*C*⋯O1	0.96	2.32	2.968 (3)	124
C35—H35*A*⋯O1	0.96	2.35	2.996 (3)	124
C24—H24*B*⋯*Cg*2^i^	0.96	2.97	3.877 (3)	158
C26—H26*A*⋯*Cg*1^ii^	0.96	2.89	3.798 (2)	157

Symmetry codes: (i) 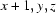; (ii) 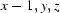.

**Fig. 1.**
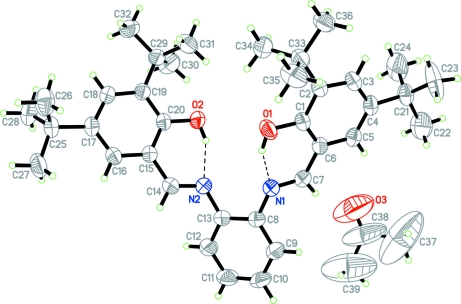
The asymmetric unit of the title compound, showing 50% probability displacement ellipsoids and the atomic numbering. Hydrogen bonds are shown as dashed lines.

**Fig. 2.**
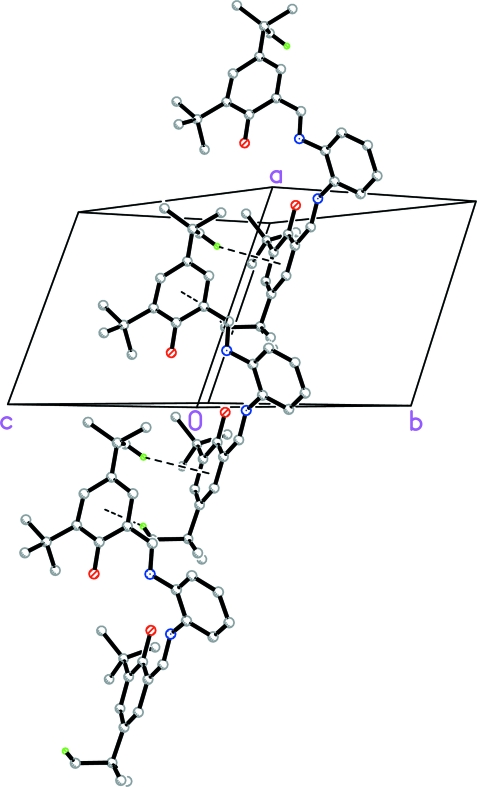
Part of the crystal packing of the title compound, showing a C—H···π bonded (dashed lines) chain along the a axis. For clarity, the solvent molecules and H atoms not involved in the interactions have been omitted.

## supplementary crystallographic information

### Comment

Schiff base compounds have received much attention because of their potential applications. Some of these compounds exhibit various pharmacological activities, such as anticancer (Dao *et al.*, 2000), anti-HIV (Sriram *et al.*, 2006), antibacterial and antifungal (Karthikeyan *et al.*, 2006) properties. In addition, some of them may be used as analytical reagents for the determination of trace elements (Eltayeb & Ahmed, 2005*a*,b). Recently, we have reported the crystal structures of 2,2'-[1,2-phenylenebis(nitrilomethylidyne)]bis(5-methylphenol) (Eltayeb *et al.*, 2007*a*) and 6,6'-dimethyl-2,2'-[1,2-phenylenebis(nitrilomethylidyne)]diphenol (Eltayeb *et al.*, 2007*b*). In this paper, we report the crystal structure of the title compound, obtained by the reaction of *o*-phenylenediamine and 3,5-di-*tert*-butylsalicylaldehyde.

In the molecular structure of the title compound, the central benzene ring (C8–13) makes dihedral angles of 46.64 (10)° and 49.34 (10)°, respectively, with the two outer benzene rings, C1—C6 and C15—C20. The dihedral angle between the two outer benzene rings is 39.13 (8)°. The C8–N1–C7–C6 and C13–N2–C14–C15 torsion angles are 178.16 (15)° and -178.34 (16)°, respectively. Bond lengths and angles in the title compound are in normal ranges (Allen *et al.*, 1987) and are comparable to those in related structures (Eltayeb *et al.*, 2007; Eltayeb *et al.*, 2007*a*,b).

The two intramolecular O—H···O hydrogen bonds, O1—H1O1···N1 and O2—H1O2···N2, generate S(6) ring motifs (Bernstein *et al.*, 1995). In addition, intramolecular C—H···O interactions (Table 1) are observed.

In the crystal structure, the acetone molecule is linked to the Schiff base molecule *via* a C—H···O hydrogen bond. The crystal structure is further stabilized by C—H···π interactions involving the C1–C6 (centroid *Cg*1) and C15–C20 (centroid *Cg*2) rings, which link the molecules into a chain along the *a* axis.

### Experimental

The title compound was synthesized by adding 3,5-di-*tert*-butylsalicylaldehyde (0.936 g, 4 mmol) to a solution of *o*-phenylenediamine (0.216 g, 2 mmol) in ethanol 95% (20 ml). The mixture was refluxed with stirring for 30 min. The resultant yellow solution was filtered. The yellow precipitate obtained after a few days, was dissolved in acetone (20 ml). Orange single crystals suitable for X-ray diffraction were formed after 6 d of slow evaporation of the acetone at room temperature.

### Refinement

Hydroxyl H atoms were located in a difference map and isotropically refined. The remaining H atoms were positioned geometrically and allowed to ride on their parent atoms, with C—H distances in the range 0.93–0.96 Å. The *U*_iso_ values were constrained to be 1.5*U*_eq_ of the carrier atom for methyl H atoms and 1.2*U*_eq_ for the remaining H atoms. A rotating group model was used for the methyl groups.

### Crystal data

**Table:**

C_36_H_48_N_2_O_2_·C_3_H_6_O	*Z* = 2
*M**_r_* = 598.84	*F*_000_ = 652
Triclinic, *P*1	*D*_x_ = 1.077 Mg m^−^^3^
Hall symbol: -P 1	Mo *K*α radiation λ = 0.71073 Å
*a* = 10.0008 (2) Å	Cell parameters from 8384 reflections
*b* = 12.0020 (3) Å	θ = 1.9–27.5º
*c* = 17.0366 (4) Å	µ = 0.07 mm^−^^1^
α = 82.068 (1)º	*T* = 296 (2) K
β = 86.320 (1)º	Block, orange
γ = 65.764 (1)º	0.59 × 0.55 × 0.43 mm
*V* = 1846.76 (7) Å^3^	

### Data collection

**Table:**

Bruker SMART APEX2 CCD area-detecto diffractometer	8384 independent reflections
Radiation source: fine-focus sealed tube	5874 reflections with *I* > 2σ(*I*)
Monochromator: graphite	*R*_int_ = 0.022
Detector resolution: 8.33 pixels mm^-1^	θ_max_ = 27.5º
*T* = 296(2) K	θ_min_ = 1.9º
ω scans	*h* = −11→12
Absorption correction: multi-scan(SADABS; Bruker, 2005)	*k* = −15→15
*T*_min_ = 0.962, *T*_max_ = 0.972	*l* = −22→22
26090 measured reflections	

### Refinement

**Table:**

Refinement on *F*^2^	Secondary atom site location: difference Fourier map
Least-squares matrix: full	Hydrogen site location: inferred from neighbouring sites
*R*[*F*^2^ > 2σ(*F*^2^)] = 0.060	H atoms treated by a mixture of independent and constrained refinement
*wR*(*F*^2^) = 0.183	*w* = 1/[σ^2^(*F*_o_^2^) + (0.085*P*)^2^ + 0.5109*P*] where *P* = (*F*_o_^2^ + 2*F*_c_^2^)/3
*S* = 1.04	(Δ/σ)_max_ = 0.001
8384 reflections	Δρ_max_ = 0.45 e Å^−^^3^
419 parameters	Δρ_min_ = −0.28 e Å^−^^3^
Primary atom site location: structure-invariant direct methods	Extinction correction: none

### Special details

**Table:**

Geometry. All e.s.d.'s (except the e.s.d. in the dihedral angle between two l.s. planes) are estimated using the full covariance matrix. The cell e.s.d.'s are taken into account individually in the estimation of e.s.d.'s in distances, angles and torsion angles; correlations between e.s.d.'s in cell parameters are only used when they are defined by crystal symmetry. An approximate (isotropic) treatment of cell e.s.d.'s is used for estimating e.s.d.'s involving l.s. planes.
Refinement. Refinement of *F*^2^ against ALL reflections. The weighted *R*-factor *wR* and goodness of fit *S* are based on *F*^2^, conventional *R*-factors *R* are based on *F*, with *F* set to zero for negative *F*^2^. The threshold expression of *F*^2^ > σ(*F*^2^) is used only for calculating *R*-factors(gt) *etc*. and is not relevant to the choice of reflections for refinement. *R*-factors based on *F*^2^ are statistically about twice as large as those based on *F*, and *R*- factors based on ALL data will be even larger.

### Fractional atomic coordinates and isotropic or equivalent isotropic displacement parameters (Å^2^)

**Table:**

	*x*	*y*	*z*	*U*_iso_*/*U*_eq_	
O1	0.24853 (14)	0.02581 (13)	0.23910 (8)	0.0551 (3)	
O2	−0.02994 (14)	0.37492 (14)	0.24879 (8)	0.0560 (4)	
N1	0.25681 (16)	0.14200 (13)	0.10087 (8)	0.0460 (3)	
N2	−0.01847 (16)	0.32660 (14)	0.10401 (8)	0.0459 (3)	
C1	0.38900 (17)	0.00930 (15)	0.24923 (10)	0.0407 (4)	
C2	0.45915 (18)	−0.04982 (14)	0.32187 (10)	0.0413 (4)	
C3	0.60129 (18)	−0.06050 (15)	0.32878 (10)	0.0449 (4)	
H3A	0.6489	−0.0987	0.3765	0.054*	
C4	0.67862 (18)	−0.01843 (15)	0.26978 (11)	0.0451 (4)	
C5	0.60684 (19)	0.03771 (16)	0.19953 (11)	0.0460 (4)	
H5A	0.6550	0.0666	0.1586	0.055*	
C6	0.46270 (18)	0.05235 (15)	0.18822 (10)	0.0421 (4)	
C7	0.3908 (2)	0.11843 (16)	0.11444 (10)	0.0449 (4)	
H7A	0.4441	0.1448	0.0753	0.054*	
C8	0.19331 (19)	0.21018 (16)	0.02816 (9)	0.0447 (4)	
C9	0.2644 (2)	0.1850 (2)	−0.04413 (11)	0.0614 (5)	
H9A	0.3569	0.1211	−0.0455	0.074*	
C10	0.1993 (3)	0.2537 (2)	−0.11419 (11)	0.0704 (6)	
H10A	0.2480	0.2363	−0.1623	0.084*	
C11	0.0627 (3)	0.3476 (2)	−0.11230 (11)	0.0668 (6)	
H11A	0.0196	0.3952	−0.1592	0.080*	
C12	−0.0113 (2)	0.37196 (19)	−0.04127 (11)	0.0575 (5)	
H12A	−0.1044	0.4354	−0.0406	0.069*	
C13	0.05201 (19)	0.30244 (16)	0.02944 (9)	0.0438 (4)	
C14	−0.1540 (2)	0.34565 (17)	0.11173 (10)	0.0489 (4)	
H14A	−0.2026	0.3410	0.0683	0.059*	
C15	−0.23510 (19)	0.37409 (16)	0.18511 (10)	0.0449 (4)	
C16	−0.38016 (19)	0.38473 (17)	0.18917 (11)	0.0489 (4)	
H16A	−0.4213	0.3742	0.1448	0.059*	
C17	−0.46321 (18)	0.41034 (15)	0.25708 (11)	0.0445 (4)	
C18	−0.39582 (18)	0.42637 (15)	0.32176 (10)	0.0439 (4)	
H18A	−0.4512	0.4449	0.3679	0.053*	
C19	−0.25311 (18)	0.41677 (15)	0.32213 (10)	0.0417 (4)	
C20	−0.17137 (18)	0.38860 (16)	0.25182 (10)	0.0424 (4)	
C21	0.8335 (2)	−0.03120 (18)	0.28575 (13)	0.0549 (5)	
C22	0.9056 (3)	0.0030 (4)	0.20973 (19)	0.1070 (11)	
H22A	0.9107	−0.0499	0.1711	0.160*	
H22B	1.0028	−0.0068	0.2212	0.160*	
H22C	0.8485	0.0870	0.1892	0.160*	
C23	0.9286 (3)	−0.1632 (3)	0.3171 (3)	0.1184 (13)	
H23A	0.8956	−0.1825	0.3693	0.178*	
H23B	1.0287	−0.1732	0.3194	0.178*	
H23C	0.9218	−0.2174	0.2826	0.178*	
C24	0.8236 (3)	0.0569 (3)	0.3431 (2)	0.0993 (10)	
H24A	0.7817	0.0362	0.3923	0.149*	
H24B	0.7628	0.1394	0.3212	0.149*	
H24C	0.9199	0.0513	0.3524	0.149*	
C25	−0.62039 (19)	0.41811 (17)	0.26502 (12)	0.0504 (4)	
C26	−0.6241 (3)	0.3137 (2)	0.32638 (16)	0.0759 (7)	
H26A	−0.5579	0.2361	0.3104	0.114*	
H26B	−0.7218	0.3169	0.3304	0.114*	
H26C	−0.5950	0.3226	0.3770	0.114*	
C27	−0.6760 (2)	0.4069 (2)	0.18631 (15)	0.0714 (6)	
H27A	−0.6773	0.4737	0.1477	0.107*	
H27B	−0.7733	0.4100	0.1935	0.107*	
H27C	−0.6122	0.3301	0.1683	0.107*	
C28	−0.7245 (2)	0.5417 (2)	0.29181 (15)	0.0668 (6)	
H28A	−0.7242	0.6076	0.2529	0.100*	
H28B	−0.6925	0.5502	0.3417	0.100*	
H28C	−0.8220	0.5447	0.2975	0.100*	
C29	−0.1868 (2)	0.43580 (17)	0.39531 (10)	0.0487 (4)	
C30	−0.1413 (3)	0.5437 (2)	0.37445 (14)	0.0736 (6)	
H30A	−0.2253	0.6167	0.3564	0.110*	
H30B	−0.0679	0.5251	0.3333	0.110*	
H30C	−0.1022	0.5571	0.4206	0.110*	
C31	−0.0546 (3)	0.3177 (2)	0.42414 (13)	0.0756 (7)	
H31A	0.0179	0.2971	0.3827	0.113*	
H31B	−0.0856	0.2516	0.4381	0.113*	
H31C	−0.0133	0.3304	0.4697	0.113*	
C32	−0.2966 (2)	0.4672 (2)	0.46406 (11)	0.0639 (5)	
H32A	−0.3809	0.5410	0.4477	0.096*	
H32B	−0.2516	0.4797	0.5082	0.096*	
H32C	−0.3263	0.4007	0.4794	0.096*	
C33	0.3806 (2)	−0.09823 (17)	0.38899 (10)	0.0485 (4)	
C34	0.2432 (2)	0.0077 (2)	0.41472 (13)	0.0718 (6)	
H34A	0.2702	0.0686	0.4316	0.108*	
H34B	0.1960	−0.0233	0.4578	0.108*	
H34C	0.1770	0.0441	0.3709	0.108*	
C35	0.3409 (3)	−0.1970 (2)	0.36056 (14)	0.0717 (6)	
H35A	0.2795	−0.1620	0.3148	0.108*	
H35B	0.2896	−0.2264	0.4021	0.108*	
H35C	0.4290	−0.2643	0.3470	0.108*	
C36	0.4771 (2)	−0.15914 (19)	0.46241 (11)	0.0597 (5)	
H36A	0.5021	−0.0995	0.4828	0.090*	
H36B	0.5651	−0.2258	0.4481	0.090*	
H36C	0.4245	−0.1902	0.5023	0.090*	
O3	0.6258 (5)	0.2364 (5)	0.0147 (2)	0.227 (2)	
C37	0.8191 (10)	0.1323 (9)	−0.0542 (7)	0.345 (8)	
H37A	0.8795	0.1561	−0.0245	0.517*	
H37B	0.8476	0.1354	−0.1090	0.517*	
H37C	0.8309	0.0501	−0.0342	0.517*	
C38	0.6661 (8)	0.2165 (6)	−0.0462 (2)	0.176 (3)	
C39	0.5935 (11)	0.2473 (7)	−0.1217 (3)	0.266 (5)	
H39A	0.5220	0.3310	−0.1262	0.399*	
H39B	0.5456	0.1933	−0.1254	0.399*	
H39C	0.6646	0.2381	−0.1637	0.399*	
H1O1	0.221 (3)	0.072 (2)	0.1925 (15)	0.083 (8)*	
H1O2	0.001 (3)	0.353 (2)	0.2023 (16)	0.084 (8)*	

### Atomic displacement parameters (Å^2^)

**Table:**

	*U*^11^	*U*^22^	*U*^33^	*U*^12^	*U*^13^	*U*^23^
O1	0.0384 (7)	0.0755 (9)	0.0528 (7)	−0.0286 (6)	−0.0115 (6)	0.0104 (6)
O2	0.0366 (7)	0.0876 (10)	0.0494 (7)	−0.0283 (7)	0.0041 (6)	−0.0191 (7)
N1	0.0396 (8)	0.0530 (8)	0.0396 (7)	−0.0134 (7)	−0.0015 (6)	−0.0038 (6)
N2	0.0390 (8)	0.0555 (8)	0.0398 (7)	−0.0151 (7)	0.0017 (6)	−0.0090 (6)
C1	0.0317 (8)	0.0428 (8)	0.0479 (9)	−0.0149 (7)	−0.0043 (7)	−0.0050 (7)
C2	0.0369 (9)	0.0401 (8)	0.0471 (9)	−0.0157 (7)	−0.0050 (7)	−0.0041 (7)
C3	0.0379 (9)	0.0432 (9)	0.0523 (9)	−0.0144 (7)	−0.0097 (8)	−0.0040 (7)
C4	0.0312 (8)	0.0437 (9)	0.0605 (10)	−0.0134 (7)	−0.0016 (8)	−0.0124 (7)
C5	0.0356 (9)	0.0499 (9)	0.0530 (10)	−0.0180 (8)	0.0053 (7)	−0.0088 (7)
C6	0.0359 (9)	0.0441 (8)	0.0444 (8)	−0.0136 (7)	−0.0004 (7)	−0.0073 (7)
C7	0.0420 (9)	0.0495 (9)	0.0412 (8)	−0.0169 (8)	0.0051 (7)	−0.0068 (7)
C8	0.0452 (10)	0.0494 (9)	0.0367 (8)	−0.0163 (8)	−0.0004 (7)	−0.0055 (7)
C9	0.0559 (12)	0.0681 (12)	0.0441 (10)	−0.0087 (10)	0.0054 (9)	−0.0112 (9)
C10	0.0746 (15)	0.0860 (15)	0.0364 (9)	−0.0185 (13)	0.0076 (9)	−0.0103 (9)
C11	0.0787 (15)	0.0719 (13)	0.0367 (9)	−0.0194 (12)	−0.0073 (9)	0.0030 (8)
C12	0.0537 (11)	0.0595 (11)	0.0466 (10)	−0.0104 (9)	−0.0061 (9)	−0.0029 (8)
C13	0.0446 (10)	0.0488 (9)	0.0372 (8)	−0.0175 (8)	−0.0008 (7)	−0.0074 (7)
C14	0.0427 (10)	0.0597 (10)	0.0431 (9)	−0.0176 (9)	−0.0041 (8)	−0.0109 (8)
C15	0.0356 (9)	0.0530 (9)	0.0445 (9)	−0.0155 (8)	0.0001 (7)	−0.0089 (7)
C16	0.0385 (9)	0.0598 (10)	0.0487 (9)	−0.0181 (8)	−0.0047 (8)	−0.0119 (8)
C17	0.0327 (9)	0.0433 (8)	0.0558 (10)	−0.0133 (7)	−0.0003 (7)	−0.0076 (7)
C18	0.0375 (9)	0.0458 (9)	0.0466 (9)	−0.0151 (7)	0.0046 (7)	−0.0079 (7)
C19	0.0392 (9)	0.0434 (8)	0.0429 (8)	−0.0171 (7)	0.0012 (7)	−0.0063 (7)
C20	0.0336 (8)	0.0486 (9)	0.0458 (9)	−0.0170 (7)	−0.0008 (7)	−0.0068 (7)
C21	0.0337 (9)	0.0586 (11)	0.0756 (13)	−0.0206 (8)	−0.0056 (9)	−0.0100 (9)
C22	0.0550 (15)	0.169 (3)	0.113 (2)	−0.0639 (19)	0.0065 (15)	−0.014 (2)
C23	0.0403 (13)	0.0770 (17)	0.225 (4)	−0.0162 (12)	−0.0399 (18)	0.016 (2)
C24	0.0628 (16)	0.123 (2)	0.133 (2)	−0.0449 (16)	−0.0094 (16)	−0.056 (2)
C25	0.0330 (9)	0.0524 (10)	0.0653 (11)	−0.0167 (8)	−0.0002 (8)	−0.0076 (8)
C26	0.0548 (13)	0.0703 (14)	0.1036 (18)	−0.0326 (11)	−0.0001 (12)	0.0087 (13)
C27	0.0423 (11)	0.0876 (16)	0.0907 (16)	−0.0283 (11)	−0.0076 (11)	−0.0222 (13)
C28	0.0402 (11)	0.0654 (12)	0.0904 (15)	−0.0158 (10)	0.0093 (10)	−0.0183 (11)
C29	0.0464 (10)	0.0596 (10)	0.0438 (9)	−0.0237 (9)	0.0002 (8)	−0.0122 (8)
C30	0.0842 (17)	0.0917 (16)	0.0721 (14)	−0.0584 (14)	0.0134 (12)	−0.0312 (12)
C31	0.0637 (14)	0.0875 (16)	0.0606 (12)	−0.0127 (12)	−0.0173 (11)	−0.0103 (11)
C32	0.0688 (14)	0.0813 (14)	0.0479 (10)	−0.0347 (12)	0.0078 (10)	−0.0191 (10)
C33	0.0442 (10)	0.0535 (10)	0.0484 (9)	−0.0227 (8)	−0.0074 (8)	0.0037 (7)
C34	0.0514 (12)	0.0857 (15)	0.0601 (12)	−0.0141 (11)	0.0040 (10)	0.0031 (11)
C35	0.0855 (16)	0.0786 (14)	0.0700 (13)	−0.0571 (13)	−0.0198 (12)	0.0145 (11)
C36	0.0620 (13)	0.0645 (12)	0.0500 (10)	−0.0255 (10)	−0.0116 (9)	0.0062 (9)
O3	0.243 (4)	0.409 (7)	0.144 (3)	−0.231 (5)	0.094 (3)	−0.137 (3)
C37	0.305 (11)	0.372 (13)	0.507 (18)	−0.242 (10)	0.246 (12)	−0.320 (13)
C38	0.300 (7)	0.273 (6)	0.091 (3)	−0.247 (6)	0.087 (4)	−0.083 (3)
C39	0.493 (15)	0.306 (9)	0.124 (4)	−0.295 (11)	0.035 (6)	−0.019 (5)

### Geometric parameters (Å, °)

**Table:**

O1—C1	1.3548 (19)	C23—H23C	0.96
O1—H1O1	0.89 (3)	C24—H24A	0.96
O2—C20	1.354 (2)	C24—H24B	0.96
O2—H1O2	0.87 (3)	C24—H24C	0.96
N1—C7	1.280 (2)	C25—C27	1.527 (3)
N1—C8	1.415 (2)	C25—C26	1.528 (3)
N2—C14	1.280 (2)	C25—C28	1.533 (3)
N2—C13	1.419 (2)	C26—H26A	0.96
C1—C6	1.399 (2)	C26—H26B	0.96
C1—C2	1.410 (2)	C26—H26C	0.96
C2—C3	1.385 (2)	C27—H27A	0.96
C2—C33	1.529 (2)	C27—H27B	0.96
C3—C4	1.394 (3)	C27—H27C	0.96
C3—H3A	0.93	C28—H28A	0.96
C4—C5	1.376 (2)	C28—H28B	0.96
C4—C21	1.531 (2)	C28—H28C	0.96
C5—C6	1.402 (2)	C29—C32	1.529 (3)
C5—H5A	0.93	C29—C30	1.532 (3)
C6—C7	1.449 (2)	C29—C31	1.533 (3)
C7—H7A	0.93	C30—H30A	0.96
C8—C9	1.388 (2)	C30—H30B	0.96
C8—C13	1.393 (2)	C30—H30C	0.96
C9—C10	1.384 (3)	C31—H31A	0.96
C9—H9A	0.93	C31—H31B	0.96
C10—C11	1.371 (3)	C31—H31C	0.96
C10—H10A	0.93	C32—H32A	0.96
C11—C12	1.379 (3)	C32—H32B	0.96
C11—H11A	0.93	C32—H32C	0.96
C12—C13	1.391 (2)	C33—C34	1.529 (3)
C12—H12A	0.93	C33—C36	1.534 (2)
C14—C15	1.448 (2)	C33—C35	1.538 (3)
C14—H14A	0.93	C34—H34A	0.96
C15—C16	1.401 (2)	C34—H34B	0.96
C15—C20	1.402 (2)	C34—H34C	0.96
C16—C17	1.374 (2)	C35—H35A	0.96
C16—H16A	0.93	C35—H35B	0.96
C17—C18	1.402 (2)	C35—H35C	0.96
C17—C25	1.534 (2)	C36—H36A	0.96
C18—C19	1.384 (2)	C36—H36B	0.96
C18—H18A	0.93	C36—H36C	0.96
C19—C20	1.406 (2)	O3—C38	1.111 (4)
C19—C29	1.535 (2)	C37—C38	1.456 (10)
C21—C24	1.507 (3)	C37—H37A	0.96
C21—C23	1.515 (3)	C37—H37B	0.96
C21—C22	1.530 (4)	C37—H37C	0.96
C22—H22A	0.96	C38—C39	1.444 (8)
C22—H22B	0.96	C39—H39A	0.96
C22—H22C	0.96	C39—H39B	0.96
C23—H23A	0.96	C39—H39C	0.96
C23—H23B	0.96		
			
C1—O1—H1O1	105.8 (16)	C27—C25—C26	108.66 (19)
C20—O2—H1O2	104.6 (17)	C27—C25—C28	107.99 (17)
C7—N1—C8	119.31 (15)	C26—C25—C28	109.09 (18)
C14—N2—C13	119.35 (15)	C27—C25—C17	111.42 (16)
O1—C1—C6	119.97 (14)	C26—C25—C17	109.29 (15)
O1—C1—C2	119.56 (15)	C28—C25—C17	110.34 (16)
C6—C1—C2	120.47 (15)	C25—C26—H26A	109.5
C3—C2—C1	116.38 (15)	C25—C26—H26B	109.5
C3—C2—C33	122.46 (14)	H26A—C26—H26B	109.5
C1—C2—C33	121.16 (15)	C25—C26—H26C	109.5
C2—C3—C4	125.10 (15)	H26A—C26—H26C	109.5
C2—C3—H3A	117.5	H26B—C26—H26C	109.5
C4—C3—H3A	117.5	C25—C27—H27A	109.5
C5—C4—C3	116.75 (16)	C25—C27—H27B	109.5
C5—C4—C21	123.03 (17)	H27A—C27—H27B	109.5
C3—C4—C21	120.18 (16)	C25—C27—H27C	109.5
C4—C5—C6	121.45 (17)	H27A—C27—H27C	109.5
C4—C5—H5A	119.3	H27B—C27—H27C	109.5
C6—C5—H5A	119.3	C25—C28—H28A	109.5
C1—C6—C5	119.85 (15)	C25—C28—H28B	109.5
C1—C6—C7	121.48 (15)	H28A—C28—H28B	109.5
C5—C6—C7	118.57 (16)	C25—C28—H28C	109.5
N1—C7—C6	122.76 (16)	H28A—C28—H28C	109.5
N1—C7—H7A	118.6	H28B—C28—H28C	109.5
C6—C7—H7A	118.6	C32—C29—C30	107.16 (16)
C9—C8—C13	119.21 (16)	C32—C29—C31	107.54 (16)
C9—C8—N1	122.34 (16)	C30—C29—C31	110.67 (19)
C13—C8—N1	118.40 (14)	C32—C29—C19	112.08 (16)
C10—C9—C8	120.87 (18)	C30—C29—C19	109.44 (16)
C10—C9—H9A	119.6	C31—C29—C19	109.92 (16)
C8—C9—H9A	119.6	C29—C30—H30A	109.5
C11—C10—C9	119.65 (18)	C29—C30—H30B	109.5
C11—C10—H10A	120.2	H30A—C30—H30B	109.5
C9—C10—H10A	120.2	C29—C30—H30C	109.5
C10—C11—C12	120.33 (18)	H30A—C30—H30C	109.5
C10—C11—H11A	119.8	H30B—C30—H30C	109.5
C12—C11—H11A	119.8	C29—C31—H31A	109.5
C11—C12—C13	120.58 (18)	C29—C31—H31B	109.5
C11—C12—H12A	119.7	H31A—C31—H31B	109.5
C13—C12—H12A	119.7	C29—C31—H31C	109.5
C12—C13—C8	119.26 (16)	H31A—C31—H31C	109.5
C12—C13—N2	122.41 (16)	H31B—C31—H31C	109.5
C8—C13—N2	118.22 (14)	C29—C32—H32A	109.5
N2—C14—C15	122.85 (16)	C29—C32—H32B	109.5
N2—C14—H14A	118.6	H32A—C32—H32B	109.5
C15—C14—H14A	118.6	C29—C32—H32C	109.5
C16—C15—C20	119.76 (15)	H32A—C32—H32C	109.5
C16—C15—C14	118.42 (16)	H32B—C32—H32C	109.5
C20—C15—C14	121.81 (16)	C2—C33—C34	110.08 (15)
C17—C16—C15	121.63 (16)	C2—C33—C36	112.41 (15)
C17—C16—H16A	119.2	C34—C33—C36	107.34 (16)
C15—C16—H16A	119.2	C2—C33—C35	109.12 (16)
C16—C17—C18	116.59 (16)	C34—C33—C35	110.84 (18)
C16—C17—C25	123.30 (16)	C36—C33—C35	107.01 (16)
C18—C17—C25	120.08 (15)	C33—C34—H34A	109.5
C19—C18—C17	124.94 (16)	C33—C34—H34B	109.5
C19—C18—H18A	117.5	H34A—C34—H34B	109.5
C17—C18—H18A	117.5	C33—C34—H34C	109.5
C18—C19—C20	116.57 (15)	H34A—C34—H34C	109.5
C18—C19—C29	121.98 (15)	H34B—C34—H34C	109.5
C20—C19—C29	121.45 (15)	C33—C35—H35A	109.5
O2—C20—C15	119.90 (15)	C33—C35—H35B	109.5
O2—C20—C19	119.60 (15)	H35A—C35—H35B	109.5
C15—C20—C19	120.49 (16)	C33—C35—H35C	109.5
C24—C21—C23	111.8 (2)	H35A—C35—H35C	109.5
C24—C21—C22	107.3 (2)	H35B—C35—H35C	109.5
C23—C21—C22	107.7 (2)	C33—C36—H36A	109.5
C24—C21—C4	108.80 (17)	C33—C36—H36B	109.5
C23—C21—C4	110.02 (17)	H36A—C36—H36B	109.5
C22—C21—C4	111.11 (18)	C33—C36—H36C	109.5
C21—C22—H22A	109.5	H36A—C36—H36C	109.5
C21—C22—H22B	109.5	H36B—C36—H36C	109.5
H22A—C22—H22B	109.5	C38—C37—H37A	109.5
C21—C22—H22C	109.5	C38—C37—H37B	109.5
H22A—C22—H22C	109.5	H37A—C37—H37B	109.5
H22B—C22—H22C	109.5	C38—C37—H37C	109.5
C21—C23—H23A	109.5	H37A—C37—H37C	109.5
C21—C23—H23B	109.5	H37B—C37—H37C	109.5
H23A—C23—H23B	109.5	O3—C38—C39	132.8 (9)
C21—C23—H23C	109.5	O3—C38—C37	116.6 (8)
H23A—C23—H23C	109.5	C39—C38—C37	110.2 (6)
H23B—C23—H23C	109.5	C38—C39—H39A	109.5
C21—C24—H24A	109.5	C38—C39—H39B	109.5
C21—C24—H24B	109.5	H39A—C39—H39B	109.5
H24A—C24—H24B	109.5	C38—C39—H39C	109.5
C21—C24—H24C	109.5	H39A—C39—H39C	109.5
H24A—C24—H24C	109.5	H39B—C39—H39C	109.5
H24B—C24—H24C	109.5		
			
O1—C1—C2—C3	−178.29 (15)	C15—C16—C17—C18	0.6 (3)
C6—C1—C2—C3	0.8 (2)	C15—C16—C17—C25	−177.69 (16)
O1—C1—C2—C33	1.3 (2)	C16—C17—C18—C19	−0.9 (3)
C6—C1—C2—C33	−179.58 (15)	C25—C17—C18—C19	177.48 (15)
C1—C2—C3—C4	−0.5 (2)	C17—C18—C19—C20	−0.1 (3)
C33—C2—C3—C4	179.91 (16)	C17—C18—C19—C29	179.91 (16)
C2—C3—C4—C5	−0.1 (3)	C16—C15—C20—O2	178.13 (16)
C2—C3—C4—C21	177.60 (16)	C14—C15—C20—O2	−0.6 (3)
C3—C4—C5—C6	0.4 (2)	C16—C15—C20—C19	−1.6 (3)
C21—C4—C5—C6	−177.27 (16)	C14—C15—C20—C19	179.70 (16)
O1—C1—C6—C5	178.51 (15)	C18—C19—C20—O2	−178.41 (15)
C2—C1—C6—C5	−0.6 (2)	C29—C19—C20—O2	1.6 (2)
O1—C1—C6—C7	2.0 (2)	C18—C19—C20—C15	1.3 (2)
C2—C1—C6—C7	−177.09 (15)	C29—C19—C20—C15	−178.68 (16)
C4—C5—C6—C1	0.0 (3)	C5—C4—C21—C24	107.0 (2)
C4—C5—C6—C7	176.58 (16)	C3—C4—C21—C24	−70.5 (2)
C8—N1—C7—C6	178.16 (15)	C5—C4—C21—C23	−130.1 (2)
C1—C6—C7—N1	0.5 (3)	C3—C4—C21—C23	52.3 (3)
C5—C6—C7—N1	−176.03 (16)	C5—C4—C21—C22	−10.9 (3)
C7—N1—C8—C9	45.8 (3)	C3—C4—C21—C22	171.5 (2)
C7—N1—C8—C13	−136.81 (18)	C16—C17—C25—C27	−5.8 (2)
C13—C8—C9—C10	2.9 (3)	C18—C17—C25—C27	176.03 (17)
N1—C8—C9—C10	−179.8 (2)	C16—C17—C25—C26	114.3 (2)
C8—C9—C10—C11	−0.2 (4)	C18—C17—C25—C26	−63.9 (2)
C9—C10—C11—C12	−1.5 (4)	C16—C17—C25—C28	−125.7 (2)
C10—C11—C12—C13	0.6 (4)	C18—C17—C25—C28	56.1 (2)
C11—C12—C13—C8	2.0 (3)	C18—C19—C29—C32	−0.8 (2)
C11—C12—C13—N2	178.2 (2)	C20—C19—C29—C32	179.21 (16)
C9—C8—C13—C12	−3.7 (3)	C18—C19—C29—C30	−119.52 (19)
N1—C8—C13—C12	178.86 (17)	C20—C19—C29—C30	60.5 (2)
C9—C8—C13—N2	180.00 (18)	C18—C19—C29—C31	118.74 (19)
N1—C8—C13—N2	2.6 (3)	C20—C19—C29—C31	−61.3 (2)
C14—N2—C13—C12	45.9 (3)	C3—C2—C33—C34	119.17 (19)
C14—N2—C13—C8	−137.91 (18)	C1—C2—C33—C34	−60.4 (2)
C13—N2—C14—C15	−178.34 (16)	C3—C2—C33—C36	−0.4 (2)
N2—C14—C15—C16	−175.52 (17)	C1—C2—C33—C36	179.98 (16)
N2—C14—C15—C20	3.2 (3)	C3—C2—C33—C35	−118.98 (18)
C20—C15—C16—C17	0.6 (3)	C1—C2—C33—C35	61.4 (2)
C14—C15—C16—C17	179.36 (17)		

### Hydrogen-bond geometry (Å, °)

**Table:**

*D*—H···*A*	*D*—H	H···*A*	*D*···*A*	*D*—H···*A*
O1—H1O1···N1	0.89 (2)	1.76 (3)	2.5809 (19)	153 (3)
O2—H1O2···N2	0.87 (3)	1.78 (3)	2.5956 (19)	155 (3)
C7—H7A···O3	0.93	2.58	3.460 (6)	158
C30—H30B···O2	0.96	2.36	2.994 (3)	123
C31—H31A···O2	0.96	2.34	2.989 (3)	124
C34—H34C···O1	0.96	2.32	2.968 (3)	124
C35—H35A···O1	0.96	2.35	2.996 (3)	124
C24—H24B···Cg2^i^	0.96	2.97	3.877 (3)	158
C26—H26A···Cg1^ii^	0.96	2.89	3.798 (2)	157

Symmetry codes:  (i) *x*+1, *y*, *z*; (ii) *x*−1, *y*, *z*.
